# The Long-Lasting Protective Effect of HGF in Cardiomyoblasts Exposed to Doxorubicin Requires a Positive Feed-Forward Loop Mediated by Erk1,2-Timp1-Stat3

**DOI:** 10.3390/ijms21155258

**Published:** 2020-07-24

**Authors:** Simona Gallo, Martina Spilinga, Elena Casanova, Alessandro Bonzano, Carla Boccaccio, Paolo Maria Comoglio, Tiziana Crepaldi

**Affiliations:** 1 Candiolo Cancer Institute, FPO-IRCCS, SP142, Km3.95, 10060 Candiolo, TO, Italy; simona.gallo@ircc.it (S.G.); elena.casanova@ircc.it (E.C.); alessandro.bonzano@ircc.it (A.B.); carla.boccaccio@ircc.it (C.B.); pcomoglio@gmail.com (P.M.C.); 2 Department of Oncology, University of Turin, Regione Gonzole 10, 10043 Orbassano, TO, Italy; martina.spilinga@ircc.it

**Keywords:** Met receptor, HGF, Erk1,2, Stat3, Timp1, anthracycline, cardiac injury, DNA damage, apoptosis

## Abstract

Previous studies showed that the hepatocyte growth factor (HGF)–Met receptor axis plays long-lasting cardioprotection against doxorubicin anti-cancer therapy. Here, we explored the mechanism(s) underlying the HGF protective effect. DNA damage was monitored by histone H2AX phosphorylation and apoptosis by proteolytic cleavage of caspase 3. In doxorubicin-treated H9c2 cardiomyoblasts, the long-lasting cardioprotection is mediated by activation of the Ras/Raf/Mek/Erk (extracellular signal-regulated kinase 1,2) signaling pathway and requires Stat3 (signal transducer and activator of transcription 3) activation. The HGF protection was abrogated by the Erk1,2 inhibitor, PD98059. This translated into reduced Y705 phosphorylation and impaired nuclear translocation of Stat3, showing crosstalk between Erk1,2 and Stat3 signaling. An array of 29 cytokines, known to activate Stat3, was interrogated to identify the molecule(s) linking the two pathways. The analysis showed a selective increase in expression of the tissue inhibitor of metalloproteinases-1 (Timp1). Consistently, inhibition in cardiomyoblasts of Timp1 translation by siRNAs blunted both Stat3 activation and the cardioprotective effect of HGF. Thus, Timp1 is responsible for the generation of a feed-forward loop of Stat3 activation and helps cardiomyocytes to survive during the genotoxic stress induced by anthracyclines.

## 1. Introduction

Hepatocyte growth factor (HGF) is a pleiotropic cytokine that regulates cell proliferation, survival, motility, scattering, morphogenesis and repair in tissue injury [1]. HGF binds the tyrosine-kinase receptor encoded by the *MET* gene. HGF binding to Met results in receptor homodimerization and autophosphorylation of two tyrosine residues (Y1234 and Y1235) located within the catalytic loop of the tyrosine kinase domain [2]. Subsequently, two tyrosines (Y1349 and Y1356) in the carboxy-terminal tail become phosphorylated and form a tandem SH2 recognition motif unique to Met [3]. When these tyrosines become phosphorylated, they recruit signaling effectors leading to activation of the Ras-Raf-Mek-Erk (extracellular signal-regulated kinase 1,2), Pi3K-Akt and Stat3 (signal transducer and activator of transcription 3) pathways [1]. HGF protects cardiac cells from hypoxic injury [4]. Silencing the *Met* gene in cardiomyocytes enhances the age-induced accumulation of reactive oxygen species [5]. Furthermore, HGF stimulates the migration and proliferation of cardiac stem cells [6].

Anthracycline doxorubicin (Doxo) is a powerful anti-cancer drug, which, however, has adverse effects on cardiac cells. The cardiotoxic activity of Doxo is due to multiple molecular mechanisms, including interaction with iron, generation of reactive oxygen species (ROS), alteration of calcium homeostasis and inhibition of beta topoisomerase II [7]. Beta topoisomerase II, highly expressed in the myocardium, forms a ternary cleavage complex with doxorubicin and DNA, inducing genotoxic stress, activation of DNA damage response (DDR) and cell death [8,9]. Recently, we found that activation of the HGF receptor, Met, alerts cardiac cells to counteract Doxo-mediated cardiotoxicity [10]. The Met-driven cardioprotection involves, at least in vitro, the activation of Stat3 [10].

Here, we show that the HGF-driven cardioprotection in H9c2 cardiomyoblasts requires Erk1,2 activation. Furthermore, inhibition of the Erk1,2 pathway abrogates the delayed activation of Stat3 at Y705 by HGF, highlighting a crosstalk between Erk1,2 and Stat3 signaling. We demonstrate that this regulatory circuit requires the tissue inhibitor of metalloproteinases-1 (Timp1). HGF upregulates Timp1 in an Erk1,2-dependent way, and in turn, Timp1 reinforces the HGF-driven activation of Stat3. Together, these results point to the existence of a positive Erk1,2-Stat3 feed-forward loop mediated by Timp1 as a new cardioprotective mechanism against doxorubicin toxicity.

## 2. Results

### 2.1. Activation of Erk1,2 by HGF Protects Cardiomyoblasts against Doxo-Induced DNA Damage and Apoptosis

**Figure 1:**
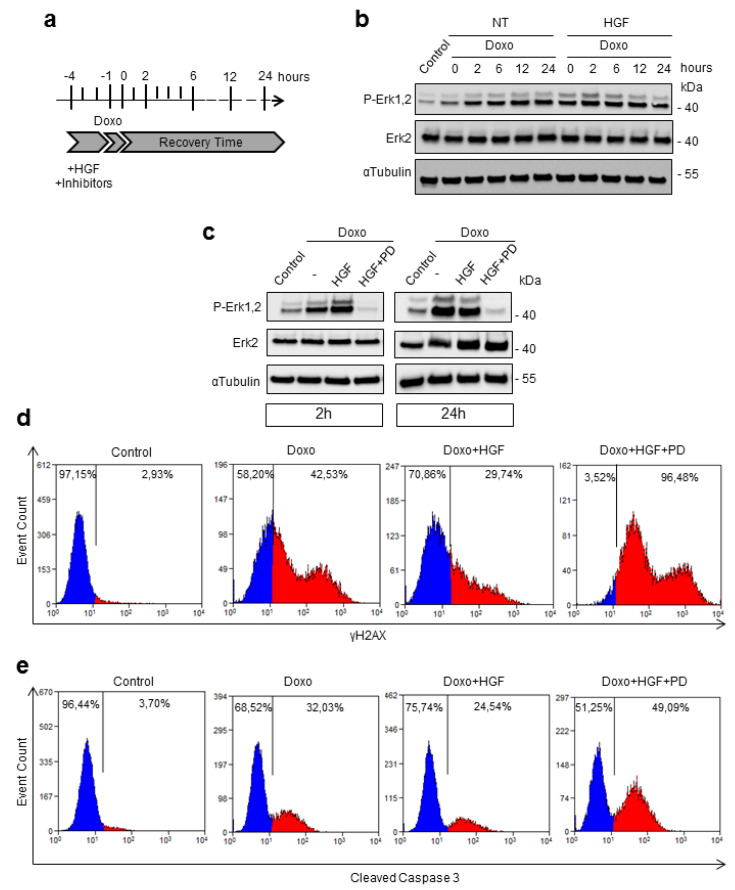
Hepatocyte growth factor (HGF) protects cardiomyoblasts against doxorubicin (Doxo)-induced DNA damage and apoptosis and the inhibition of Erk1,2 (extracellular signal-regulated kinase 1,2) dampens this cardioprotection. (**a**) Scheme of the experimental protocol used for treatments. H9c2 cardiomyoblasts were incubated with Doxo (25 μM) for 1 h (Doxo pulse) or pretreated for 4 h with HGF (0.5 nM) alone or HGF+PD (PD98059, Erk inhibitor) and exposed to Doxo in the last hour. Then, the cells were replaced with fresh low serum medium (recovery time). (**b**) Control, Doxo- and Doxo+HGF-treated cells were analyzed at different time points of the recovery time (0, 2, 6 12 and 24 h). (**c**) Control, Doxo-, Doxo+HGF- and Doxo+HGF+PD-treated cells were analyzed at 2 and 24 h of recovery time. P-Erk1,2 and total Erk2 proteins were detected by Western blotting. αtubulin was used as the loading control in all Western blots. (**d**) The level of γH2AX protein was analyzed by flow cytometry. (**e**) The level of cleaved caspase 3 protein was analyzed by flow cytometry. Data are representative results of three independent experimental replicates.

Evidence from the literature indicates that Erk1,2 is a prosurvival factor associated with resistance to chemotherapy in cancer cells [11,12,13]. We therefore addressed whether Erk1,2 signaling, downstream of Met, could be involved in the adaptation response to doxorubicin. H9c2 cardiomyoblasts, an immortalized cell line derived from embryonic rat heart [14], were pulsed with Doxo for 1 h and allowed to recover for a further 24 h (Figure 1a). Erk1,2 phosphorylation at TEY sites (T202/Y204 in Erk1 and T183/185 in Erk2) increased immediately after Doxo pulse stimulation (0 time) and was persistent up to 24 h post-drug treatment (Figure 1b). Pretreatment with HGF for 4 h further increased the P-Erk1,2 levels after Doxo pulse stimulation, which however returned to Doxo-induced basal levels within 6 h (Figure 1b). This suggests that priming with HGF increases the responsiveness of the Erk pathway.

Next, we addressed the role of Erk1,2 in the response to DNA damage. It is known that doxorubicin, in complex with beta topoisomerase II and DNA, induces double-strand breaks, which in turn activate a strong stress response known as DDR [15]. Persistent DDR signaling stimulates histone H2AX phosphorylation on S139 (γH2AX), a sensitive marker of genotoxic stress. One hour exposure to Doxo, followed by 24 h recovery, stimulated a strong DDR, as shown by increased levels of γH2AX analyzed by flow cytometry measurements (Figure 1d). Pretreatment with HGF significantly attenuated the DDR response (Figure 1d). Pharmacological inactivation of Erk1,2 by the specific chemical inhibitor, PD98059, administrated during HGF pretreatment, blocked the phosphorylation of Erk1,2 at TEY sites (Figure 1c) and, importantly, dampened the HGF protection against the genotoxic stress (Figure 1d). Sustained Doxo-induced DNA damage triggers cell death by stimulation of the apoptotic pathway, involving the activation of the caspase cascade [16]. One hour exposure to Doxo, followed by 24 h recovery, triggered a strong increase in cleaved caspase 3, a generally accepted marker of apoptosis, as shown by flow cytometry measurements (Figure 1e). HGF pretreatment significantly reduced the level of Doxo-induced increase in cleaved caspase 3 (Figure 1e). Importantly, pharmacological inactivation of Erk1,2 by PD98059 abolished the HGF-driven protection from apoptosis (Figure 1e).

### 2.2. A Crosstalk Exists between Erk1,2 and Stat3 in Met-Mediated Cardioprotection

**Figure 2:**
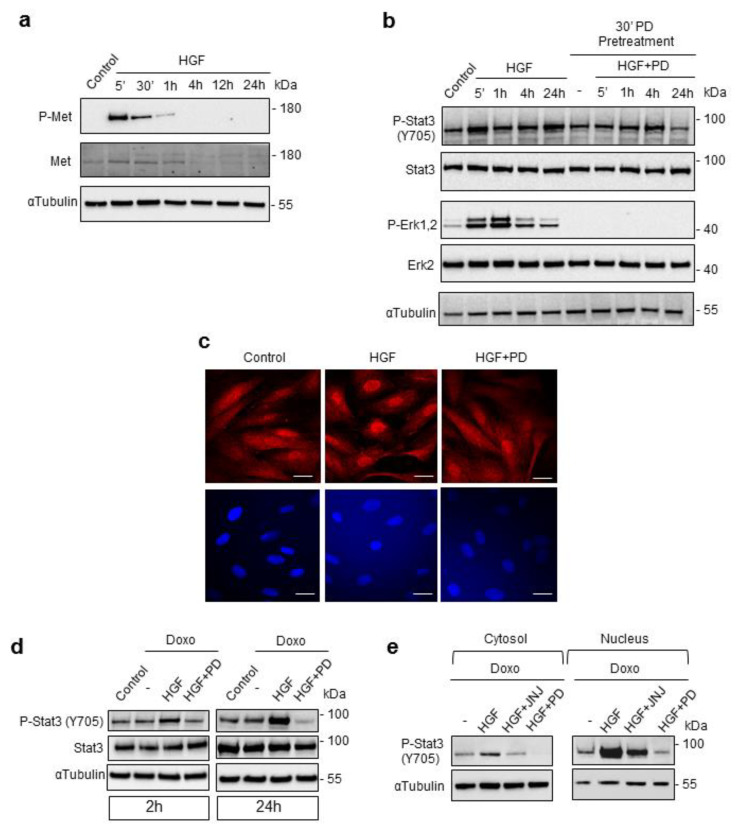
Crosstalk between Erk1,2 and Stat3 (signal transducer and activator of transcription 3) is established in H9c2 cardiomyoblasts. (**a**,**b**) H9c2 cells were untreated (control) or treated with HGF (0.5 nM) for different lengths of time; (**b**) cells were also treated with HGF+PD (PD98059, 1 µM). Proteins were detected by Western blotting with specific antibodies: (**a**) P-Met and total Met; (**b**) P-Stat3 (Y705), total Stat3, P-Erk1,2 and total Erk2. (**c**) Stat3 protein localization was analyzed by immunofluorescence after HGF treatment for 24 h. Representative images of Stat3 (top) and Dapi (bottom) staining are shown. Bar: 10 μm. (**d**,**e**) H9c2 cells were untreated (control) or treated with Doxo (25 μM), Doxo+HGF (0.5 nM), Doxo+HGF+PD (PD98059, 1 µM) and (**e**) Doxo+HGF+JNJ (JNJ38877605 Met inhibitor, 500 nM). For cell treatments, see Figure 1a. Cells were treated with the inhibitors (PD and JNJ) for 4 h and exposed to Doxo in the last hour. Protein levels of P-Stat3 (Y705) and total Stat3 were detected by Western blotting at 2 h (**d**) and 24 h (**d**,**e**) recovery time after the end of Doxo treatment. (**e**) P-Stat3 (Y705) protein levels were evaluated in the cytosol and nuclear fractions. αtubulin was used as the loading control in all Western blots. Data are representative results of three independent experimental replicates.

We have previously shown that in the presence of Stattic, a Stat3 inhibitor, preconditioning with HGF failed to protect against Doxo-induced genotoxicity and apoptosis [10]. We observed a long-lasting activation of Stat3. In previous work, Stat3 was found to be transiently phosphorylated by HGF at early times [17]. We evaluated the kinetics of HGF-Met autophosphorylation and downstream Stat3 and Erk1,2 pathways in H9c2 cardiomyoblasts. Met was strongly phosphorylated at Y1234-Y1235 after 5 and 30 min post-HGF stimulation. This autophosphorylation was followed by subsequent Met protein downregulation (Figure 2a). Stat3 phosphorylation on Y705 was rapidly induced by HGF (after 5′), and then returned to basal level after 1 h. Interestingly, the second wave of strong Stat3 phosphorylation on Y705 was observed 24 h of HGF stimulation (Figure 2b). Phosphorylation of Erk1,2 at TEY sites significantly increased after 5 min of HGF stimulation, remained high for 1 h and then dephosphorylated after 4 h of HGF treatment (Figure 2b). The delayed HGF-induced Stat3 phosphorylation occurred after a wave of Erk1,2 activation, suggesting the existence of crosstalk between the two pathways. In line with this idea, the delayed phosphorylation of Stat3 on Y705 at 24 h was blunted by the PD98059 inhibitor (Figure 2b). These results were paralleled by the increased nuclear staining of Stat3 after prolonged HGF stimulation, which was reduced in the presence of the PD98059 inhibitor (Figure 2c). In Doxo-treated cells, Stat3 phosphorylation on Y705 was comparable to control starved cells (Figure 2d). When cells were pretreated with HGF, enhanced Stat3 phosphorylation was found at late times (2 and 24 h recovery time, Figure 2d) and was blunted by the PD98059 inhibitor also in Doxo-treated cells (Figure 2d). The nuclear translocation of Stat3 was dampened by the specific Met (JNJ-38877605) and Erk1,2 (PD98059) inhibitors (Figure 2e).

### 2.3. HGF-Met-Erk1,2 Preconditioning Leads to Stimulation of Timp1 Protein Synthesis Followed by Stimulation of Stat3 Signaling

**Figure 3:**
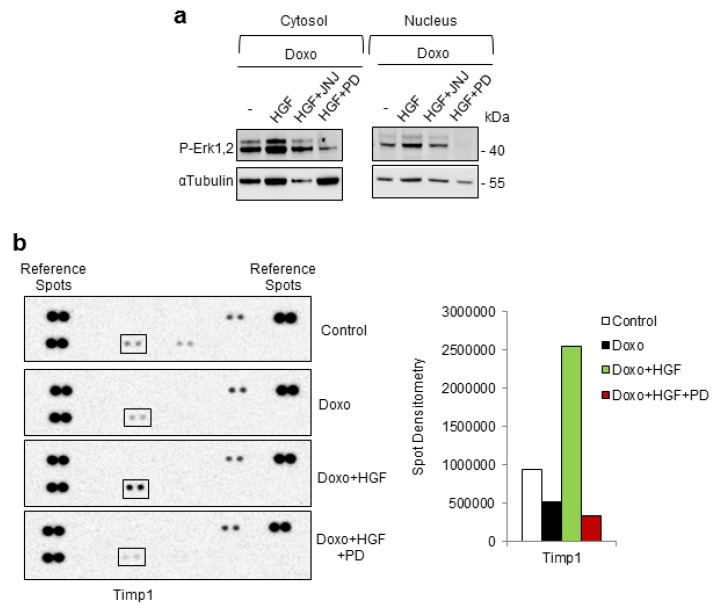
Cytokine profiles induced by HGF-Met-Erk1,2 preconditioning against Doxo damage. H9c2 cells were untreated (control) or treated with Doxo (25 μM), Doxo+HGF (0.5 nM), Doxo+HGF+PD (PD98059, 1 µM) (**a**,**b**) and Doxo+HGF+JNJ ((JNJ38877605, 500 nM) (**a**). Cells were treated with the inhibitors (PD and JNJ) for 4 h and exposed to Doxo in the last hour. For cell treatments, see Figure 1a. (**a**) P-Erk1,2 protein levels were detected in cytosol and nuclear fractions. αtubulin was used as the loading control. (**b**) Representative images (left) and densitometric analysis (right) of protein samples that were probed with the rat cytokine antibody array, that allowed analyzing 29 cytokines simultaneously. Data are representative results of three independent experimental replicates.

Next, we challenged the hypothesis that the HGF-stimulated Erk1,2 signaling could generate a feed-forward loop of Stat3 activation through autocrine/paracrine secretion of cytokines (Figure 3). Figure 3a shows that phospho-Erk1,2 was translocated to the nucleus by HGF to an extent higher than Doxo alone. This was confirmed by co-treatment with HGF and the specific Met inhibitor JNJ-38877605 (Figure 3a). Inhibition by PD98059 during HGF treatment blocked the nuclear translocation of phospho-Erk1,2 in the context of Doxo treatment (Figure 3a). These data suggested that HGF-mediated Erk1,2 activation could imply a transcriptional mechanism in the regulation of Stat3 activation. The next step was to identify putative cytokines involved in the cardioprotective action exerted by the HGF-stimulated Erk1,2 pathway. We, thus, used an array-based proteomic screen to detect different cytokines simultaneously (Table A1). The rat cytokine antibody array is coated with specific antibodies against 29 different cytokines, among which are known Stat3-activating cytokines, such as IL-6, IL-10 and TNF-α (tumor necrosis factor alpha). H9c2 cells were untreated or treated with Doxo, Doxo+HGF and Doxo+HGF+PD (PD98059 inhibitor). Protein samples were probed with the array and the relative cytokine levels were evaluated (Figure 3b). Compared with untreated samples, there was an increase in the levels of Timp1 cytokine in cells pretreated with HGF (Doxo+HGF, Figure 3b). Interestingly, this cytokine was downregulated by the Erk1,2 inhibitor (Doxo+HGF+PD, Figure 3b).

**Figure 4:**
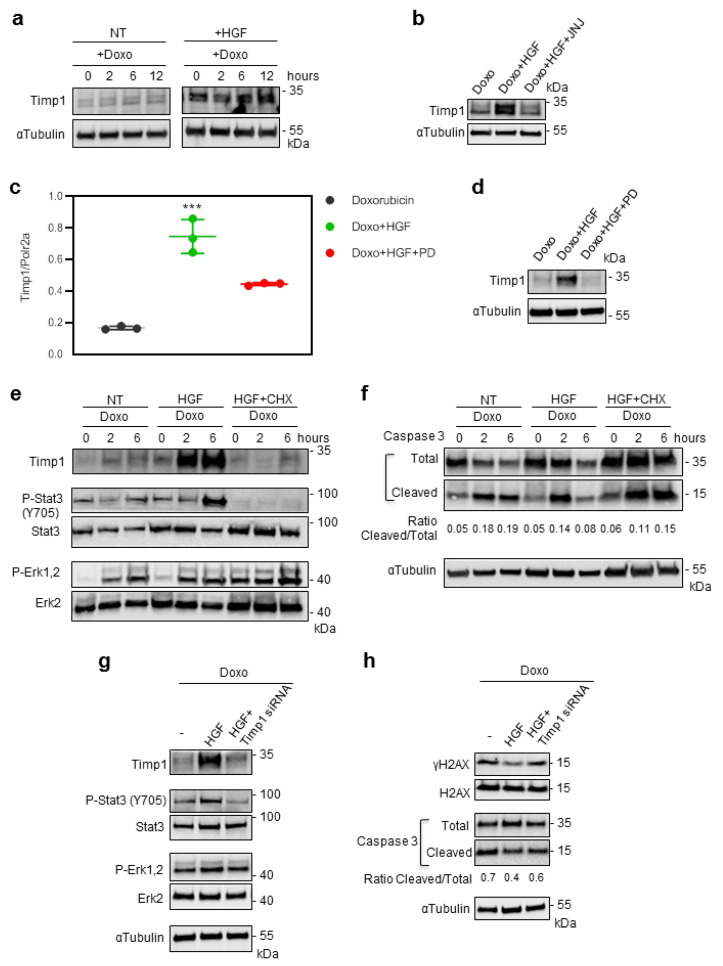
Preconditioning with HGF induces Timp1 (tissue inhibitor of metalloproteinases-1) protein synthesis, which is followed by Stat3 phosphorylation and protection against Doxo-induced apoptosis. (**a**) H9c2 cardiomyoblasts were treated with Doxo (25 μM) or Doxo+HGF (0.5 nM). For cell treatments, see Figure 1a. Timp1 protein levels were evaluated by Western blotting at different points of recovery time (**a**). Cells were also treated with (**b**) 500 nM JNJ38877605 Met inhibitor (Doxo+HGF+JNJ) or (**c**,**d**) 1 µM PD98059 Erk1,2 inhibitor (Doxo+HGF+PD). Protein (**b**,**d**) and mRNA (**c**) levels of Timp1 were analyzed after 24 h of recovery time. Polr2a was used as reference gene for the expression data normalization. *** *p* < 0.005 significantly different from Doxo-treated cells. (**e**,**f**) H9c2 cells were pretreated with HGF alone, or with HGF+Cycloheximide (CHX, 10 μM) for 4 h. Then, Doxo (25 μM) was added in the last 1 h. The CHX treatment was performed 30 min before adding HGF and was maintained during all the treatment protocol. Protein levels of Timp1, P-Stat3 (Y705), Stat3, P-Erk1,2, Erk2 (**e**) and total and cleaved caspase 3 (**f**) were detected at different time points of recovery time (0, 2 and 6 h). (**g**,**h**) H9c2 cells were treated with Doxo (25 μM), Doxo+HGF (0.5 nM) or Doxo+HGF+Timp1 siRNA. Protein levels of Timp1, P-Stat3 (Y705), Stat3, P-Erk1,2 and Erk2 (**g**) and γH2AX, H2AX and cleaved/total caspase 3 ratios (**h**) were measured after 24 h of recovery time. The ratios calculated between cleaved and total caspase 3 are shown. αtubulin was used as the loading control in all Western blots. Data are representative results of three independent experimental replicates.

To validate the cytokine array results, we evaluated the Timp1 protein and mRNA levels in H9c2 cells treated with Doxo and Doxo+HGF (Figure 4). HGF pretreatment led to an increase in Timp1 protein levels starting from the end of Doxo pulse (0 h) and these high levels were maintained until 24 h (Figure 4a,b,d). HGF pretreatment produced also an increase in Timp1 mRNA levels (Figure 4c). The effect of HGF on Timp1 was blunted by both Met (JNJ-38877605)- and Erk (PD98059)-specific inhibitors (Figure 4b–d, respectively), demonstrating that the HGF effect on Timp1 protein expression is a specific Met-Erk1,2-driven mechanism. Accordingly, by interfering with protein translation through cycloheximide (CHX), we completely blocked the HGF-mediated effect on Timp1 protein synthesis (Figure 4e). Interestingly, in the presence of CHX, HGF did not induce the tyrosine phosphorylation of Stat3, whereas Erk1,2 phosphorylation was maintained (Figure 4e). The treatment with CHX also impaired the apoptosis protection mediated by HGF pretreatment (Figure 4f). Altogether, these data suggest that the long-lasting cardioprotective mechanism mediated by Stat3 might follow the HGF-Met-Erk1,2 induction of Timp1 de novo protein synthesis. To confirm the involvement of Timp1 in Met-mediated cardioprotection against Doxo, we knocked down Timp1 in H9c2 cells treated with the chemotherapeutic protocol (Figure 4g,h). The reduction in Timp1 protein levels impaired the HGF-induced Stat3 phosphorylation on Y705, whereas it did not have any effect on phospho-Erk1,2 (Figure 4g). Importantly, the Timp1 siRNA produced also the impairment of Met-mediated cardioprotection against Doxo, as indicated by the analysis of γH2AX and cleaved/total caspase 3 ratios (Figure 4h). These data suggest that HGF promotes long-lasting cardioprotection via a positive feed-forward loop mediated by Erk1,2-Timp1-Stat3.

## 3. Discussion

Doxorubicin is a potent and widely used drug for cancer treatment. However, its use is limited by the cumulative dose-dependent cardiotoxicity. In the present study, we show that: (i) Erk1,2 is activated in response to doxorubicin; (ii) HGF stimulation enhances the responsiveness of Erk1,2 signaling; (iii) pharmacological inhibition of Erk1,2 blunts the HGF-mediated cardioprotection against doxorubicin, suggesting that the Erk1,2 signaling is an anti-genotoxic and pro-survival pathway stimulated by the Met receptor.

A previous study by Navarro et al. [18] involved superoxide anions in Erk1,2 stimulation by doxorubicin in hepatocytes. Although the mechanism(s) of Erk1,2 activation by environmental stresses are not yet clear, the Erk transduction system is widely exploited by cancer cells in chemotherapy resistance [13,19,20]. Erk1,2 signaling is also an important pro-survival signaling pathway in the heart [21]. Erk1,2 belongs to the so-called reperfusion injury salvage kinase (RISK) pathway, which confers cardioprotection when activated specifically at the time of reperfusion and ROS production [22]. Doxorubicin cardiotoxicity is due to multiple molecular mechanisms, including the generation of ROS, which further contributes to genotoxic stress, caused by its complex with DNA and beta topoisomerase II [8]. In line with these studies, our data show that the Erk1,2 pathway is needed also in cardiomyocytes in the adaptive response to anthracycline-induced cardiotoxicity. HGF stimulation results in enhanced defense against the pro-apoptotic and genotoxic effects promoted by doxorubicin in cardiomyocytes [10]. Here, we implicate an important role for Erk1,2 signaling in the adaptive response against doxorubicin DNA damage and apoptosis.

We previously showed that activation of Met by HGF alleviates doxorubicin-induced genotoxicity and apoptosis via Stat3 [10]. Evidence from the literature indicate that Stat3 is activated by epidermal growth factor receptor (EGFR) stress-exposed tumor cells and is associated with a survival advantage for tumor cells (reviewed in Balanis and Carlin [23]). In line with this, Stat3 is also implicated in pro-survival signaling cascades activated upon ischemia/reperfusion [24]. Stat3 belongs to the so-called survivor activating factor enhancement (SAFE) pathway, another powerful protective pathway that involves the activation of TNFα and JAK, and protects against reperfusion injury when given as a preconditioning stimulus [25].

In the present study, we showed that the delayed strong increase in Stat3 phosphorylation on Y705, promoted by HGF, requires Erk1,2 activation. Stat3 Y705 is phosphorylated by Met and its dimerization and activation occur following tyrosine phosphorylation [17]. Our results suggest a new mechanism of Stat3 phosphorylation, involving a positive feed-forward loop of activation promoted by Erk1,2. The cytokine array used in our study contained many Stat3-activating cytokines, such as IL-6, IL-10 and TNF-α. Surprisingly, there were no differences in these and other cytokine contents among cells treated with Doxo, Doxo+HGF and Doxo+PD except for Timp1. These data suggest that the long-lasting cardioprotective mechanism mediated by Stat3 might follow the HGF-Met-Erk1,2 induction of Timp1 de novo protein synthesis. Consistently, inhibition in cardiomyoblasts of Timp1 translation by cycloheximide or three siRNAs blunted both Stat3 activation and the cardioprotective effect of HGF. Timp1 is an inhibitor of matrix metalloproteinases, which are capable of degrading most components of the extracellular matrix. Timp1 was among the most (more than six-fold) significantly upregulated genes found in mice with cardiomyocyte-specific expression of activated Met [26]. Importantly, Timp1 is an inhibitor of the Met sheddase Adam10 (a disintegrin and metalloproteinase-10), leading to the accumulation of Met at the cell surface and enhancement of Met signaling in liver metastasis [27]. Adam10 is expressed in H9c2 cardiomyoblasts (data not shown). Thus, it is possible that also in cardiac cells, the Timp1 cytokine may reduce HGF-induced Met shedding, producing an enduring downstream signal. Alternatively, Timp1 may favor the interaction of Met with other cell membrane receptor(s), which may address Met to perinuclear endosomes, where Stat3 is protected from inactivating protein tyrosine phosphatases and remains phosphorylated at Y705 [28]. A possible candidate is integrin β1, which forms a complex with Met and drives metastasis and invasive resistance in tumor cells [29]. Moreover, Timp1 has been recognized as an antiapoptotic/pro-survival cytokine interacting with specific surface receptors that initiate intracellular signaling [30]. Further investigation will address these possibilities.

However, HGF as a cardioprotective mediator could be a double-edged sword. HGF is involved in cancer progression, and thus tumors driven by Met alterations (amplification and/or overexpression) could exploit HGF to develop and progress [1]. In addition, Timp1 is upregulated and associated with poor clinical outcome for several cancers [31]. Further investigation in oncological models will address whether HGF-Timp1-based preventive cardioprotective therapy could lead to a tumor-protecting effect in the context of anthracycline chemotherapy. In addition, from a clinical point of view, cancer patients identified for HGF-based preventive cardioprotective therapy should be selected on the basis of a precise analysis of Met genetic lesions.

**Figure 5:**
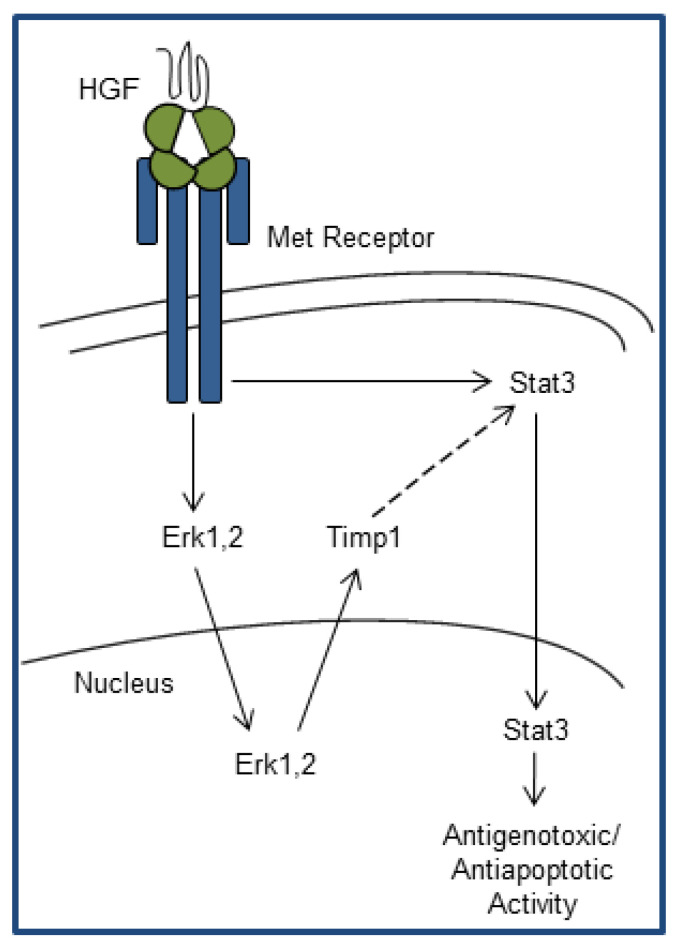
Model of the cardioprotective action against anthracycline damage mediated by Met-Erk1,2-Timp1-Stat3 signaling in cardiac cells. HGF treatment stimulates the Met receptor that quickly leads to phosphorylation and nuclear translocation of Erk1,2. In the nucleus, Erk1,2 induces gene transcription of Timp1, the inhibitor of metalloproteases, which allows an endurable Met-dependent activation of Stat3. Thus, Met belatedly stimulates Stat3 and exerts cardioprotective functions. Black arrow lines indicate phosphorylation/activation. Dotted arrow line indicates activation within the cascade.

In conclusion, the present study provides evidence that Timp1 protein expression is upregulated by a Met-Erk1,2-dependent mechanism and provides a new connection between Erk and Stat3 signaling downstream of the HGF receptor (Figure 5). Thus, Met activation and Timp1 upregulation may help cardiomyocytes survive in conditions of stress, as it happens in organ injury by anthracyclines.

## 4. Materials and Methods

Reagents: The rat cardiomyoblasts cell line H9c2 was purchased from the American Type Culture Collection (CRL1446, Manassas, VA, USA). HGF was acquired from Tebu-Bio (Le-Perray-en-Yvelines, FR). Doxorubicin Hydrochloride (100-39, Pfizer, New York, NY, USA) was obtained from the Pharmacy of Candiolo Cancer Institute, FPO-IRCCS. Met tyrosine kinase inhibitor JNJ-38877605 (JNJ) was kindly provided by Janssen Pharmaceutica (Beerse, Belgium). Mek1,2 inhibitor PD98059 was purchased from Sigma-Aldrich (19-143, St. Louis, MO, USA). Cycloheximide solution (C7698, CHX) was purchased from Sigma-Aldrich.

Cell culture and treatments: H9c2 cells were cultured in Dulbecco’s modified Eagle’s medium (D5671, Sigma-Aldrich) supplemented with 10% fetal bovine serum (10270-098, Gibco, Thermo Fisher Scientific, Waltham, MA, USA), 1% penicillin, 1% streptomycin (P0781, Sigma-Aldrich) and 1% L-Glutamine (G7513, Sigma-Aldrich) and were incubated under 5% CO2 at 37  °C. Cells were passed regularly and subcultured to ~80/90% of confluence. At 24 h before the onset of the treatment, cells were cultured in low serum medium (0.5% fetal bovine serum). Doxo was used at 25 μM for 1 h, a dose leading to full-blown DNA damage and apoptosis. HGF (0.5 nM) and the inhibitors PD98059 (PD, 1μM) and JNJ-38877605 (JNJ, 500 nM) were administered 3 h before Doxo treatment. Then, the cells were maintained with fresh low serum medium for a further 24 h (scheme of Doxo treatment is shown in Figure 1a).

Western blot analyses: H9c2 cells (*n* = 3 per group) were lysed in ice-cold RIPA lysis buffer added with a protease inhibitor cocktail (2714, Sigma-Aldrich). Lysates were subsequently sonicated and centrifuged at 12,000× *g* at +4 °C for 20 min. The cytosol and nuclear fractions were produced by the NE-PER Nuclear and Cytoplasmic Extraction Reagents following the manufacturer’s protocol (78833, Thermo Fisher Scientific). The protein concentration was evaluated with the BCA Protein Assay Kit (23225, Thermo Fisher Scientific). Proteins and the pre-stained protein ladder (10–180 kDa, 26616, PageRuler Thermo Fisher Scientific) were separated by electrophoresis using precast 4–12% SDS-PAGE gels (NP0321, Invitrogen, Carlsbad, CA, USA) and transferred to a Hybond-P PVDF membrane (1704158, Bio-Rad, Hercules, CA, USA). After incubation in blocking solution (10% bovine serum albumin, BSA, A2153, Sigma-Aldrich) at room temperature, membranes were incubated overnight at +4  °C with the primary antibodies: P-Met, 3077, Cell Signaling (Danvers, Massachusetts, United States); Met, AF527, R&D; P-Erk1,2, 4376, Cell Signaling; Erk2, sc-154, Santa Cruz; P-Stat3, Y705, 9145, Cell Signaling; Stat3, 9139, Cell Signaling; Timp1, AF580, R&D; cleaved caspase 3, 9579, Cell Signaling; γH2AX, 9718, Cell Signaling; H2AX, 2595, Cell Signaling. Primary antibodies were diluted in BSA 5% TBS-Tween and re-used at most three times. Membranes were washed and then incubated with specific horseradish peroxidase-conjugated secondary antibodies (115-035-003, 111-035-144, 705-035-003, Jackson Laboratory, Bar Harbor, ME, USA) for 1 h at room temperature. Secondary antibodies were diluted in TBS-Tween and used once only. The proteins were revealed by enhanced chemiluminescence of the ECL Prime detection kit and quantified with the Image Lab software (170-5061, Bio-Rad). The data shown are the representative results of the three independent experimental replicates.

Flow cytometric analysis: H9c2 cells (*n* = 3 per group) were treated with FIX & PERM reagents (GAS-002, ADG Wien, Austria) and then stained for 30 min at room temperature, in the dark, with, separately, the anti-γH2AX (Ser139, 9718, Cell Signaling) rabbit antibody (9718, Cell Signaling) and the anti-cleaved Caspase-3 rabbit antibody (559565, BD Pharmingen, San Diego, CA, USA). Then, secondary antibody incubation was performed with the anti-rabbit IgG (H+L) APC (4050-11S, Southern Biotech, Birmingham AL) for 30 min at room temperature, in the dark. Samples were analyzed on a CyAn ADP 9-color analyzer (Beckman Coulter, Brea, CA, USA). The data shown are the representative results of the three independent experimental replicates.

Immunofluorescence analysis: Cells (*n* = 3 per group) were plated in Fibronectin (F0895, 3μg/mL, Sigma-Aldrich)-coated 24-well plates, fixed with ice-cold 100% Methanol (32213, Sigma-Aldrich) for 10 min at −20 °C and washed with PBS. Fixed cells were permeabilized with 0.1% Triton X-100 (X100, Sigma-Aldrich). Then, the cells were saturated with 5% normal serum (566460, Sigma-Aldrich) and incubated overnight at +4 °C with the anti-Stat3 primary antibody (9139, Cell Signaling). Secondary antibody incubation was performed with the Alexa Fluor 555-conjugated goat anti-rabbit antibody (A27039, Invitrogen) for 1 h at room temperature. DNA was counterstained with DAPI, added at the end of secondary antibody incubation for 15 min at room temperature. Both primary and secondary antibodies were diluted in 1% BSA 0.3%-Triton X-100 and used once only. Immunofluorescence images were taken by the Leica TCS SP2 AOBS confocal laser-scanning microscope and processed with the LAS AF software (Leica Microsystems). The data shown are the representative results of the three independent experimental replicates.

Rat cytokine antibody arrays: To detect simultaneously different cytokines, we exploited a rat cytokine antibody array allowing to analyze 29 cytokines (Rat Cytokine Array Panel A, ARY008, R&D). To perform the assay, we followed the manufacturer’s protocol.

Real-time PCR analysis: Total RNA was extracted from H9c2 cells (*n* = 3 per group) with the miRNeasy mini Kit, according to the manufacturer’s protocol (217004, Qiagen, Hilden, Germany). The extracted RNA was quantified with NanoDrop and the reverse transcription was performed with iScript Reverse Transcription Supermix, according to the kit protocol (1708841, Biorad, CA, USA). Quantitative PCR assay was performed on an ABI 7500 Fast Real-Time PCR System using the TaqMan Fast Universal PCR master mixture and TaqMan Gene Expression Assay Probes for Timp1 (Mm00441818_m1) and Polr2a (Rn01752026_m1; Applied Biosystems, Waltham, MA, USA). PCR reactions were performed in triplicate per each sample and normalized to Polr2a gene expression.

Knock-down (siRNA) experiments: H9c2 cells (*n* = 3 per group) were transfected with lipofectamine 2000 (11668019, Thermo Fisher Scientific) alone and with a pool of three Timp1 siRNAs (AM16708: 190472, 190473, 190474; Thermo Fisher Scientific) in Optimem medium (31985070, Gibco, Thermo Fisher Scientific) for 6 h. Then, the Optimem medium was changed with the DMEM supplemented with 10% fetal bovine serum and the cells were kept growing overnight. The next day, cells were cultured in low serum medium (FBS 0.5%) and treated with the chemotherapeutic protocol (see the “Cell culture and treatments” section and the scheme of Doxo treatment in Figure 1a). siRNA experiments were set using a scrambled siRNA as a negative control (Silencer Negative Control *n*°1 siRNA, AM4611, Thermofisher). To reduce the off-target effects, “Predesigned Invitrogen Silencer siRNAs” (Thermofisher) were chosen. They are designed for maximum specificity using a highly effective and extensively tested algorithm. No biological and molecular effects mediated by Timp1 pool siRNAs were found. The data shown are the representative results of the three independent experimental replicates.

Statistical analysis: All values are expressed as the mean ± standard deviation of the independent experiments. The analysis was performed on treatment groups with a sample size of at least 3 independent experiments. For multiple comparisons, one-way ANOVA analysis was used, followed by Tukey’s post hoc test. The T-test was performed when the ANOVA F value was significant (*p* < 0.05) and there was no variance inhomogeneity. The data analysis was done using the GraphPad Prism software.

## Abbreviations

DDR	DNA damage response
Doxo	Doxorubicin
Erk1,2	Extracellular signal-regulated kinase 1, 2
γH2AX	S139 phosphorylated histone H2AX
HGF	Hepatocyte growth factor
JNJ	JNJ-38877606
PD	PD98059
RISK pathway	Reperfusion injury salvage kinase pathway
ROS	Reactive oxygen species
Stat3	Signal transducer and activator of transcription 3
Timp1	Tissue inhibitor of metalloproteinases-1

## Appendix A

**Table A1:** Scheme of the rat cytokine antibody arrays used in the work. Rat Cytokine Array Panel A, 29 analyzed cytokines (R&D).

	1_2	3_4	5_6	7_8	9_10	11_12	13_14	15_16	17_18	19_20
A	Reference Spots	CINC-1	CINC-2α/β	CINC-3	CNTF	Fractalkine	GM-CSF	sICAM-1	IFN-γ	Reference Spots
B		IL-1α	IL-1β	IL-1ra	IL-2	IL-3	IL-4	IL-6	IL-10	
C		IL-13	IL-17	IP-10	LIX	L-Selectin	MIG	MIP-1α	MIP-3α	
D	Reference Spots	RANTES	Thymus Chemokine	TIMP1	TNF-α	VEGF	Negative Control			
